# Adsorption Characteristics of Sol Gel-Derived Zirconia for Cesium Ions from Aqueous Solutions

**DOI:** 10.3390/molecules19079160

**Published:** 2014-07-01

**Authors:** Sobhy M. Yakout, Hisham S. Hassan

**Affiliations:** 1 Biochemistry Department, College of Science, King Saud University, PO Box, 2455, Riyadh 11451, Kingdom of Saudi Arabia; 2 Hot Laboratories Center, Atomic Energy Authority, P. Box 13759, Cairo, Egypt

**Keywords:** cesium, zirconia, sol gel, kinetics, thermodynamics

## Abstract

Zirconia powder was synthesized via a sol gel method and placed in a batch reactor for cesium removal investigation. X-ray analysis and Fourier transform infrared spectroscopy were utilized for the evaluation of the developed adsorbent. The adsorption process has been investigated as a function of pH, contact time and temperature. The adsorption is strongly dependent on the pH of the medium whereby the removal efficiency increases as the pH turns to the alkaline range. The process was initially very fast and the maximum adsorption was attained within 60 min of contact. A pseudo-second-order model and homogeneous particle diffusion model (HPDM) were found to be the best to correlate the diffusion of cesium into the zirconia particles. Furthermore, adsorption thermodynamic parameters, namely the standard enthalpy, entropy, and Gibbs free energy, were calculated. The results indicate that cesium adsorption by zirconia is an endothermic (ΔH > 0) process and good affinity of cesium ions towards the sorbent (ΔS > 0) was observed.

## 1. Introduction

Over the past years, scientists have focused on the problem of radioactive pollution, especially since the nuclear accidents at Chernobyl and Fukushima that released a great volume of radionuclides into the environment. Cesium (Cs) is one of most important radionuclides that has many applications such as in the construction industry, radiation detection, cancer therapy, mineral processing, *etc*. [1] Cesium is present in spent nuclear fuel because it is one of the main products of nuclear fission [2]. Given its chemical similarity to alkalis, long-life, and high solubility/mobility, Cs is easy taken up by both terrestrial and aquatic animals and then bioaccumulates in the food chain [3]. In the case of cesium exposure, the body adsorbs it and it affects the renal and liver functions, causes reproductive function disorders and also thyroid cancer [4], so it is necessary to remove it from waste solutions. Numerous studies have been done to find cheap methods for Cs removal from waste solutions. A good review on adsorption removal of cesium from drinking waters was done by Liu *et al*. [5].

Recently, inorganic ion-exchangers have seen many applications in nuclear technology due to their high chemical, mechanical, and radiation stabilities and good compatibility with the final waste forms Moreover they have high ion-exchange and adsorption capacity [6]. Metal oxides are inorganic ion exchangers that are extensively used in radioactive waste treatment [7]. Metal oxides’ structural and ion exchange properties are influenced by the synthesis method [8,9,10,11,12,13,14,15]. The sol–gel process is an attractive method for inorganic ion-exchanger synthesis for many reasons: low temperature synthesis, simple equipment is used, thin film formability and so on. Adsorbent powders of metal oxide origin, synthesized by the sol-gel process in this work, have not previously been used in removing cesium from aqueous solutions. In the present study, zirconia was successfully prepared by a sol-gel technique and used for removal of Cs^+^ ions from aqueous solution. The adsorption kinetic and thermodynamics of Cs adsorption from aqueous solutions by the sol-gel derived zirconia was studied.

## 2. Results and Discussion

### 2.1. X-ray Diffraction (XRD) Analysis

**Figure 1:**
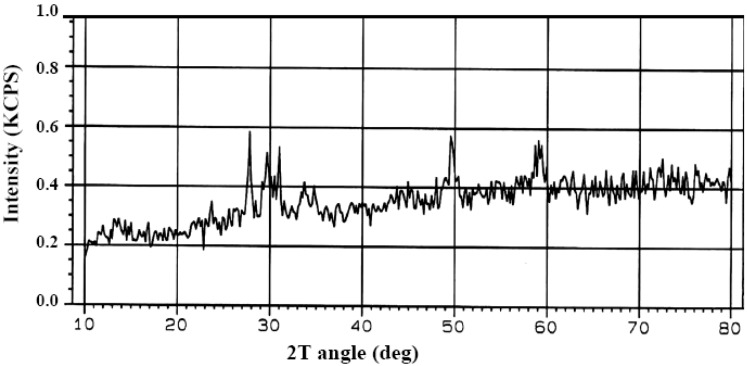
X-ray diffraction pattern of prepared zirconia at 900 °C.

X-ray diffraction is one of the techniques commonly used for the structural characterization of inorganic ion-exchangers. The X-ray diffraction (XRD) patterns using Cu Kα of the sol-gel derived zirconia are shown in Figure 1. By analyzing the XRD patterns of the synthesized materials, it was observed that X-ray pattern showed a monoclinic zirconium phase at 900 °C. The three strongest peaks (2θ ≈ 30.0, 50.0 and 60.0) could be assigned to zirconia [16], The peak at 2θ = 28, is considered due to the monoclinic phase. The sharp peak at 2θ = 30 corresponds to the tetragonal phase [17].

### 2.2. FTIR Characterization

**Figure 2:**
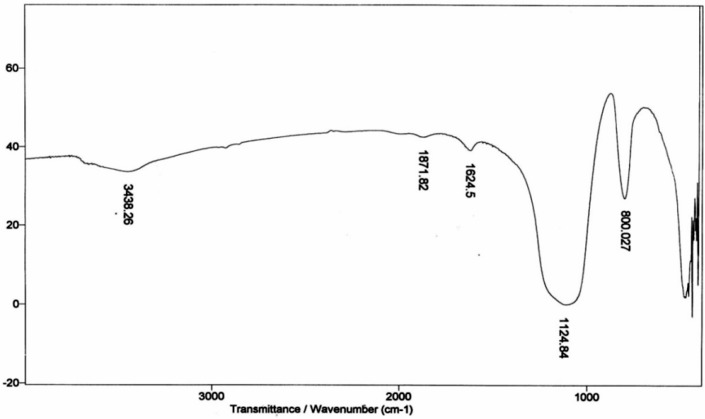
IR spectrum of prepared zirconium oxide.

The infrared spectrum of the prepared zirconia were recorded in the 200–4000 cm^−1^ range and is shown in Figure 2. The broad absorption band in the 3,396–2,950 cm^−1^ range is due to the stretching vibrations of the water molecule OH groups [18], whereas the absorption band which appears at 1,624.5 cm^−1^ is characteristic of the bending vibration of water molecules. It is uncertain whether the water observed in these spectra reflects the composition of the surface resulting from the heating process, or water which had rapidly attached to the surface during cooling. The peaks at 1,124.8 and 800 cm^−1^ are due to the bending vibration of hydroxyl groups bound to zirconium oxide [19]. 

### 2.3. Effect of pH

**Figure 3:**
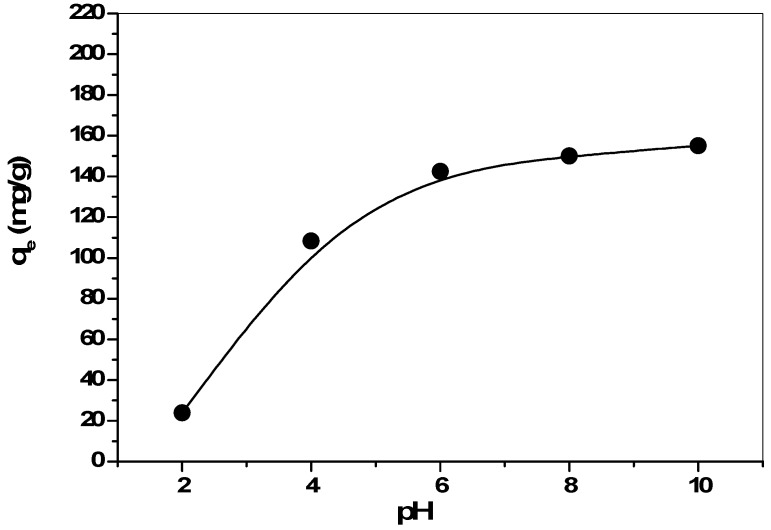
Effect of solution pH on uptake of Cs by zirconia.

It is known that pH is important factor for the adsorption of metal ions on adsorbents. Specifically it affects the solution chemistry of the solute as well as the functional groups present in the sorbent. Results of the effect of solution pH on adsorption of cesium ions on the zirconia are shown in Figure 3. The plot shows a marked influence with a gradual rise in the uptake with increase in pH from 2 to 7 and thereafter it remained constant. The variation in the removal of the Cs with respect to pH can be explained by considering the functional groups present on the surface of the zirconia and the nature of the physicochemical interaction of the species in solution. Zirconia contains hydroxyl groups which can act good role in ion-exchange reaction through the substitution of its protons by cesium ion, according to the following reactions [20]:

nCs^+^ + Zr(-OH)_m_ ↔ Zr(-O-Cs)_m−n_ + nH^+^ (1)

where -OH = hydroxyl group, nH^+^ = number of protons released, and Zr refers to the zirconia surface. In such a system, at low pH, because of the high concentration of H^+^, there is competition of excess H^+^ ions with cesium ions for binding onto the zirconia surface. Then Equation (1) lies to the left. At the same time the predominant charges on zirconia are positive, which results in the lower uptake of positively charged Cs ions on zirconia. With increased pH, active sorption sites available for Cs ions increase as a result of the deprotonation of ion exchange sites and then the negative charge on the sorbent increases. Therefore, an electrostatic attraction between the negatively charged sorbent surface and the positively charged Cs ions will occur. This means that Equation (1) proceeds further to the right and metal ion removal is increased. Within this pH range, the ion exchange process is the major mechanism for removal of metal ions from solution. In the subsequent studies, experiments were performed at a solution pH value of 7 to avoid any possible hydroxide precipitation. Further, a decrease in the solution pH was observed after equilibration as compared to the initial solution pH. The drop in equilibrium pH suggests that H^+^ ions are liberated from the solid surface into the aqueous phase as a result of the exchange with metal cations. 

### 2.4. Effect of Contact Time and Temperature

**Figure 4:**
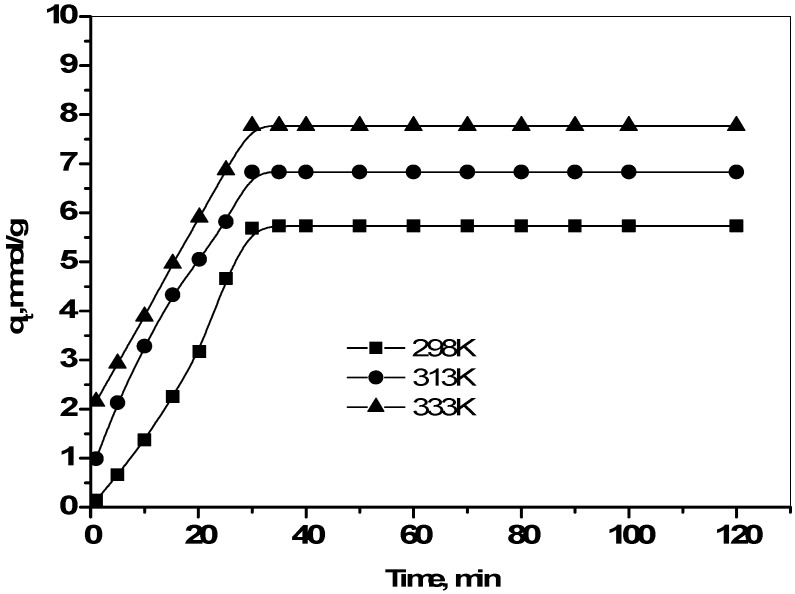
Effect of contact time on Cs^+^ ions adsorption onto prepared zirconia at 298, 313, and 333 K.

The adsorption profile of Cs uptake with time at different temperatures is shown in Figure 4. The removal curves are singular, smooth and continuous, leading to saturation, suggesting possible monolayer coverage of Cs ions on the surface of the zirconia and typically 80%–90% adsorption of the equilibrium value for each ion occurred within 30 min. Cs removal increases with time and attains equilibrium at 60 min. A short equilibrium time is one of the important considerations for economical wastewater treatment applications. The initial rapid adsorption of Cs ions on zirconia is due to the availability of a larger number of vacant adsorption sites for the Cs of the bulk solution. The subsequent slower adsorption is likely because of the competition among the Cs ions for the limited number of vacant adsorption sites. Thus the driving concentration gradient between the bulk solution and the solid surface is the main factor controlling the kinetics of the system.

The equilibrium sorption capacity of Cs onto zirconia was found to increase with increasing temperature, indicating that the Cs ion sorption on the adsorbent was favored at higher temperatures. The sorption of Cs is endothermic, thus the extent of sorption increased with increasing temperature [21]. The sorption of Cs by zirconia involves not only physical but also chemical sorption. At high temperature, ions are readily dehydrated, and therefore their sorption becomes more favorable [22].

### 2.5. Kinetic Study

**Figure 5:**
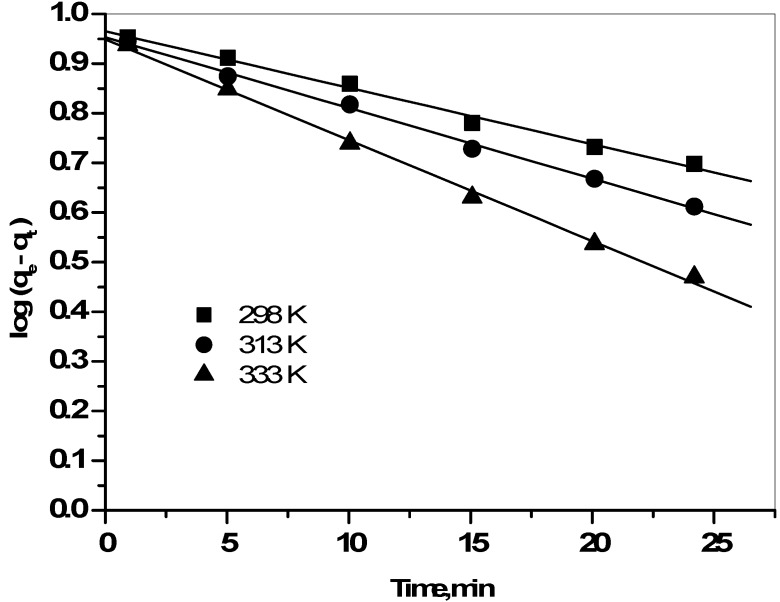
Pseudo first order plots for the sorption of Cs^+^ ions onto prepared zirconia at 298, 313, and 333 K.

**Figure 6:**
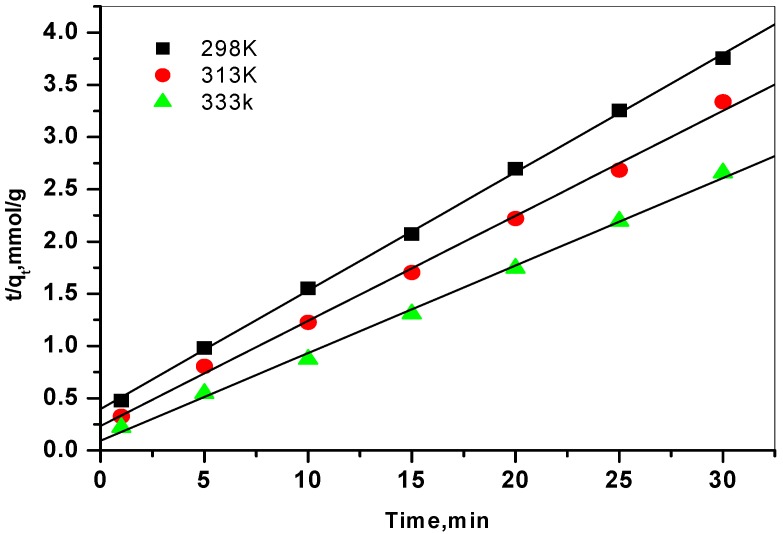
Pseudo second order plots for the sorption of Cs^+^ ions onto prepared zirconia at 298, 313, and 333K.

Analysis of experimental date at various time points makes it possible to calculate the kinetic parameters, and use some of this information for designing and modeling the adsorption processes. To understand the adsorption mechanism of zirconia for Cs, the adsorption kinetics were investigated using pseudo first-order [23], pseudo-second-order [24], with Equations (2) and (3) respectively:

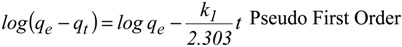
(2)

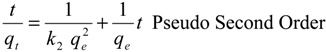
(3)
where q_t_ and q_e_ are the amount adsorbed (mmol/g) at time t and at equilibrium time, respectively and K_1_, K_2_are the first and second rate adsorption constants respectively, respectively. By testing the two plots of log(q_e_ − q_t_) *versus* t (Figure 5), and (t/q_t_) *versus* t (Figure 6), the rate constants k_1_, and k_2_, can be calculated. The conformity between experimental data and each model predicted values was expressed by the correlation coefficient (R^2^) in Table 1.

The result indicates that the pseudo-second-order model (R^2^ = 0.98) is more suitable than the pseudo first-order kinetic model (R^2^ = 0.92) for Cs adsorption on zirconia, and that the adsorption complies with the pseudo-second-order reaction kinetics. The calculated q_e_ values obtained from the first-order kinetic model do not give reasonable values, as they are too low compared with the experimental q_e_ values. Estimated q_e_ values of the pseudo-second-order model accurately predict the adsorption kinetics over the entire working times and temperatures. Therefore, this model has enough sufficiency for precise and acceptably accurate prediction of the kinetics of Cs adsorption onto zirconia. This suggested the overall rate of the adsorption process is most likely to be controlled by the chemisorption process [25] and rate of reaction is directly proportional to the number of active sites on the surface of adsorbent. From Table 1, it can be shown that the values of the initial sorption rate (h = k_2_qe^2^) increased with the increase in temperature. According to the pseudo second order model, the adsorption rate dqt/dt is proportional to the second order of (qe − qt). Since zirconia has a relatively high equilibrium adsorption density qe, the adsorption rates become very fast and the equilibrium times are short. Such short equilibrium times coupled with high adsorption capacity indicate high degree of affinity between adsorbate molecules and the carbon surface [26]. These results explain that the pseudo second order sorption mechanism is predominant and that the overall rate constant of each ion exchange process appears to be controlled by the chemical sorption process [27,28,29].

**Table 1:** The calculated parameters of the pseudo first and second order kinetic models.

Model	Parameter	Temperature, K		
		298	313	333
Experimental date	qe, exp, mmolg/g	5.9	7.1	7.9
	K_1_, g/mmolg min	0.038	0.032	0.035
Pseudo-First order	q_e_, mmolg/g	8.5	10.0	10.5
	R^2^	0.92	0.91	0.92
Pseudo-Second order	K_2,_ g/mmolg min	0.02	0.02	0.2
	q_e,_mmolg/g	7.01	8.04	9.25
	h, mmol/g min	0.94	1.09	1.76
	R^2^	0.999	0.999	0.999

One of the most widely used models to describe the kinetics of ion exchange data and predict the actual slowest step is the Homogeneous Particle Diffusion Model (HPDM). In this model, the rate-determining step of sorption normally involves two main steps: film diffusion that involves diffusion of ions through the liquid film surrounding the adsorbent and/or particle diffusion that involves diffusion of ions into the adsorbent beads. If film diffusion is the rate-determining step, the following expression can be utilized to calculate the diffusion coefficient:

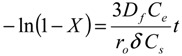
(4)
where *C_e_* and *C_r_* are the equilibrium concentrations of the cesium ion in solution and solid phases respectively, *D_f_* is the film diffusion coefficient in the liquid phase, *X* is the fraction attainment of equilibrium or extent of adsorbent conversion, *r_o_* is the radius of the adsorbent particle, *δ* is the thickness of the liquid film. If film diffusion was involved in cesium adsorption, then the plot of −ln(1 − X) *vs.* time would be a straight line passing through the origin. 

**Figure 7:**
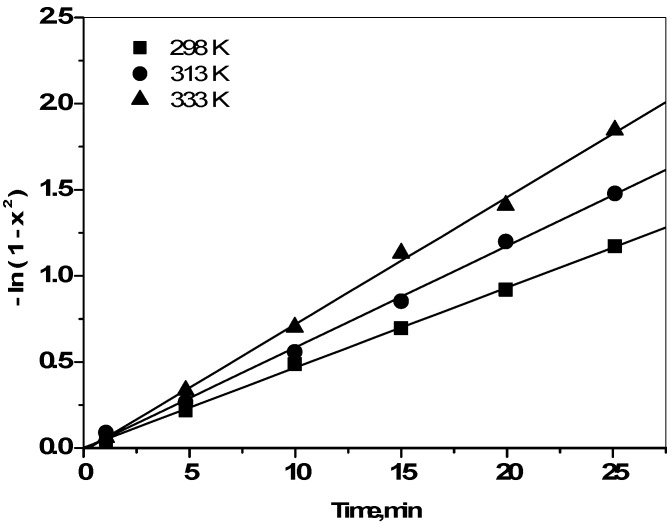
Plots of –ln(1 − x^2^) as a function of time for the diffusion of Cs^+^ ions onto prepared zirconia at 298, 313, and 333K.

If the diffusion of Cs ions through the adsorbent beads is the slowest step, the particle diffusion will be the rate determining step and the particle diffusion model can be applied to calculate the diffusion coefficients. Then, the rate equation is expressed by Equation (5):

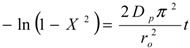
(5)
where, *D_p_* is the particle diffusion coefficient. If particle diffusion was involved in cesium adsorption, then the plot of −ln(1 − X^2^) *vs.* time would be a straight line passing through the origin. The kinetic rate data of Cs^+^ ions sorbed onto zirconia powder were tested using Equations (4) and (5). The kinetic plots of ln(1 − X) *vs.* time exhibit straight lines that do not pass through the origin for all studied temperatures. This indicates that the film diffusion model does not control the rate of the sorption processes. When adsorption starts, the reacted layer thickness is still very small and film resistance to Cs ion diffusion is therefore comparable to the adsorbent outer shell resistance. Moreover, ln(1 – X^2^) *vs.* t plots are given in Figure 7. A straight line with zero intercept would suggest cesium adsorption to be controlled by its diffusion within the particles of zirconia. 

### 2.6. Thermodynamic Studies

**Figure 8:**
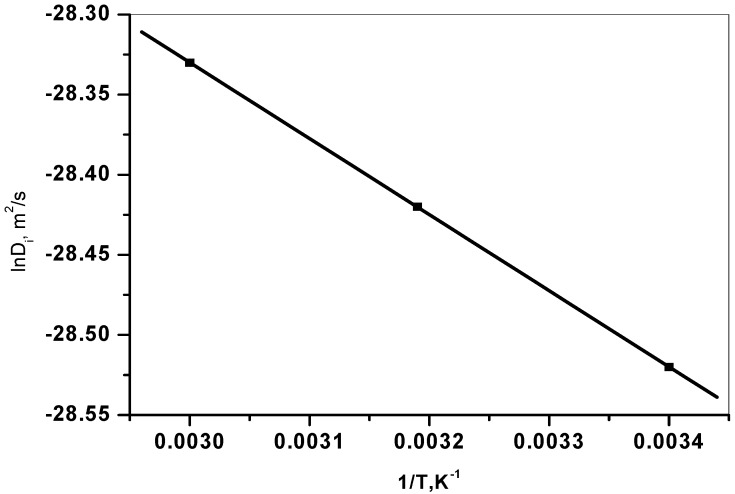
Arrhenius plot for the sorption of Cs^+^ ions onto zirconium oxide.

In any sorption process, both energy and entropy considerations must be taken into account in order to determine what processes will occur spontaneously. Values of thermodynamic parameters are the actual indicators for practical application of a process. Diffusion coefficient of cesium sorption is expressed as a function of temperature by the following Arrhenius type relationship:

ln *D_p_* = ln *D_o_* − (*E_a_* / *RT*) (6)

where, *D_o_* is a pre-exponential constant analogous to Arrhenius frequency factor. A plot of ln D_i_ *vs* 1/T was found to be linear (Figure 8). The E_a_ value calculated from the slope of the plot is equal to 4.0 kJ/mol. The relatively low activation energy (less than 42 kJ/mol) suggested that Cs sorption is a diffusion-controlled process (Kuo and Lotse [30]).

The other thermodynamic parameters, change in the free energy (ΔG), enthalpy (ΔH), and entropy (ΔS), were determined by using the following equations [31,32,33]:

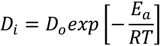
(7)

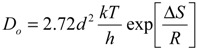
(8)

ΔG^*^ = ΔH^*^ − *T*ΔS^*^ = *E_a_* + *RT* − *T*ΔS^*^ (9)

where *k* is the Boltzmann constant, *h* is Plank’s constant, *d* is the average distance between two successive positions, *R* is the gas constant and *T* is the absolute temperature. Assuming that the value of *d* is equal to 5 × 10^−8^ cm, [34] the values of thermodynamic parameters were calculated and are presented in Table 2. The positive ΔH values indicate the presence of an energy barrier in the sorption and an endothermic process [35]. The positive value of entropy change (ΔS) reflects good affinity of cesium ions towards the sorbent and the increasing randomness at the solid-solution interface during the sorption process [36]. The positive ΔG^*^ values suggest the existence of an energy barrier and that the reaction is a non-spontaneous process [37]. 

**Table 2:** Diffusion and thermodynamic parameters of the sorption of Cs+ ions onto zirconia.

Tem. K	D_i_ 10^−11^, m^2^/s	D_o_ 10^−12^ m^2^/s	E_a_ KJ/mol	R^2^	ΔS^*^ J/mol k	ΔG^*^ KJ/mol	ΔH^*^ KJ/mol
298		4.12		0.999			
313	4.56	2.07	3.95	0.999	464.4	128.5	1.68
333		4.98		0.999			

## 3. Experimental

### 3.1. Chemicals and Reagents

All chemicals used were of analytical reagent grade. An accurately weighed quantity of cesium (I) nitrate (purchased from Merck, Darmstadt, Germany) was dissolved in deionized water to prepare a stock solution. Experimental solutions of the desired concentrations were obtained by successive dilutions. All sample bottles and glassware were cleaned; rinsed with deionized water and oven dried at 60 °C.

### 3.2. Preparation and Characterization of Zirconium Oxide

In the present work, the synthesis of zirconia was carried out using a sol-gel polymerization route. Briefly, a polymerization reaction between urea and formaldehyde was carried out at 70–80 °C with stirring for 1 h in an alkaline medium (pH 8–9) to form the respective resin. Zirconium nitrate [Zr(NO_3_)_4_] was added during the resin formation. Ethylene glycol was used to terminate the polymerization reaction. The gel produced was slowly dried at 120 °C and then calcined at 900 °C for 2 h to produce the zirconia powder [38]. 

The X-ray powder diffraction patterns of the prepared material were recorded on film at room temperature in a Philips XRG 3100 X-ray diffractometer (Philips electronic instrument, Mahwah, NJ) using Cu Kα X-ray operated at 30 Kv and 30 mA with a fixed slit. FTIR analysis was investigated by a Fourier transform infrared spectrometer (IR Prestige-21, Shimadzu, Tokyo, Japan). The thermo gravimetric analysis (TGA) was carried out using Shimadzu TGA-50 analyzer (Tokyo, Japan).

### 3.3. Sorption Studies

Zirconia powder (100 mg) in 25 mL conical flasks containing cesium solution (10 mL, 10^−2^ M) were mixed. The conical flasks then covered with aluminum foil and were then placed in a thermostatic shaker at room temperature for different time intervals. The adsorbent was finally removed by filtration, Cs concentration was determined radiometrically, using a NaI crystal using a pulse height multi-channel analyzer (McA) model 8000 obtained from Amptek (Bedford, MA, USA). Influence of solution pH on the sorption of Cs (30 ppm) was studied in the pH range of 2.0 to 10.0 under similar experimental conditions. Before each experiment, the solution pH was initially adjusted using HCl and/or NaOH depending on the required pH value. The thermodynamic studies were investigated by carrying out batch study at different temperatures. The temperatures chosen for study were 298, 313, and 333 K. The temperature of the Cs solution was adjusted using a thermostatic water bath (Model WB29, Memmert, Munich, Germany). The Cs uptake *qt* (mg/g) at any time *t* was calculated from the mass balance as follows:

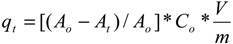
(10)
where *A_o_* and A*_t_* are the initial and time interval activities of metal ion in solution, *V* is the volume of the solution (L) and *m* is the weight of the adsorbent (g) and Co is the initial concentration (mmol/L) of the metal ion used.

## 4. Conclusions

The use of zirconia for cesium ion removal from aqueous solution was studied. The highest removal efficiency of zirconia for cesium ion was obtained at pH 7.0. The adsorption can be explained as an ion exchange mechanism between cesium ion and hydroxyl groups. The kinetic studies showed that most of the cesium ion uptake occurred rapidly in the first 30 min, and the adsorption equilibrium was reached within one hour. The adsorption kinetics were well described by a pseudo-second-order and homogeneous particle diffusion models, implying that chemisorption is a predominant mechanism and particle diffusion control cesium adsorption. Based on the values obtained from some adsorption thermodynamic parameters such as ΔH, ΔS and ΔG, it was found that cesium adsorption on zirconia is an endothermic process and cesium ions show good affinity towards the sorbent.
